# NCAPG promotes the malignant progression of endometrioid cancer through LEF1/SEMA7A/PI3K-AKT

**DOI:** 10.7150/jca.100951

**Published:** 2025-01-01

**Authors:** Zhen Ren, Xiaohan Li, Chun Fu

**Affiliations:** Department of Obstetrics and Gynecology, The Second Xiangya Hospital of Central South University, Changsha, Hunan, 410011, China.

**Keywords:** endometrioid carcinoma, NCAPG, LEF1, SEMA7A, PI3K/AKT signaling pathway

## Abstract

NCAPG promotes the progression of endometrial cancer (EC) through PI3K-AKT pathway and has potential as a novel tumor marker. However, the precise regulatory mechanism of NCAPG remains inadequately understood. In this study, we applied Assay for Transposase Accessible Chromatin with high-throughput sequencing (ATAC-Seq) analysis combined with chromatin immunoprecipitation-qPCR (CHIP) and Co-Immunoprecipitation (CoIP) analysis to analysis for the first time that NCAPG promotes EC cell proliferation, migration and invasion by affecting the binding of LEF1 to chromatin, thereby affecting the transcription of downstream SEMA7A. Mechanistically, SEMA7A regulated the PI3K-AKT signaling pathway by binding to the PI3K regulatory subunit p85, exerting its biological function. The NCAPG/LEF1/SEMA7A axis promoted EC tumorigenesis and progression by activating the PI3K-AKT signaling pathway. Additionally, LEF1 and SEMA7A were associated with the FIGO stage, pathological grade, and myometrial invasion in EC patients. The expressions of NCAPG, LEF1, and SEMA7A were highly consistent. Targeting this cascade may provide effective antitumor strategies to delay the progression of EC.

## Introduction

During mitosis or meiosis, a cell typically replicates its genetic material and distributes the newly copied chromosomes equally to each daughter cell^1^, ^2^. The cohesin complex is a protein essential for chromosome cohesion and segregation during mitosis and meiosis^3^. The core subunit of cohesin includes members that maintain chromosome structure, such as Condensin I and II. Condensin I has unique non-SMC subunits including NCAPG, NCAPH, and NCAPD2^4^. NCAPG (non-SMC condensin I complex subunit G) plays a crucial role in chromosome condensation and stabilization during meiosis and mitosis cell division^5^. NCAPG is encoded by the NY-MEL-3 gene and located on human chromosome 4p15.32, with an approximate size of 114.1 kDa^6^. Chromosomal instability (CIN), characterized by frequent chromosome missegregation, is one of the most significant genomic abnormalities in endometrial cancer (EC)^7^-^9^. Therefore, exploring the molecular mechanism behind NCAPG's cancer-promoting effects and its role in chromosome condensation in EC could lead to develop novel targeted therapy strategies against cancer cell mitosis.

Numerous studies have investigated the role of NCAPG in tumors. In colorectal cancer cells, knocking down NCAPG reduces cell proliferation ability^10^. Similarly, knockdown of NCAPG in ovarian cancer inhibits proliferation, promotes apoptosis, and induces cell cycle arrest in the G2/M phase^11^. In liver cancer cells, knockdown of NCAPG leads to S-phase cell cycle arrest by inhibiting cyclin A1 and CDK2^12^. Additionally, high NCAPG expression in prostate cancer cells enhances their migration and invasion capabilities^13^.

Previous research has established that NCAPG functions as an oncogene in EC cells, facilitating cell proliferation, invasion, and homologous recombination repair via the PI3K/AKT pathway^14^. As a crucial factor in chromatin condensation and sister chromatid separation during the cell cycle, the specific intermediary mechanism through which NCAPG affects the PI3K/AKT pathway remain to be elucidated. This study employed high-throughput transposase-accessible chromatin sequencing (ATAC-Seq) following knocking down NCAPG in the HEC-1-A cell line. ATAC-Seq is a technology designed to identify regions of open chromatin. For genes to be expressed during transcription and translation, the relevant chromatin segments must be in an open state, permitting the binding of transcription factors and other regulatory elements, a concept referred to as chromatin staining—quality accessibility. To meticulously examine how chromatin accessibility impacts gene transcription, the study integrated ATAC-Seq data with RNA-Seq results. Our findings indicate that NCAPG enhances the transcription of SEMA7A by modulating the chromatin accessibility of the transcription factor LEF1, thereby activating the PI3K-AKT pathway and promoting the proliferation, migration, and invasion of EC cells.

## Materials and Methods

### Patient Samples

Patient samples were obtained from the Department of Gynecology at the Second Xiangya Hospital of Central South University, under research ethics registration number LYF2022231. Informed consent forms were signed by all participants. The study encompassed 84 patients diagnosed with endometrioid carcinoma. The inclusion criteria were as follows: (1) patients underwent primary surgical intervention without any preoperative hormonal therapy, radiotherapy, or chemotherapy; (2) postoperative pathological analysis confirmed the diagnosis of superficial endometrioid cancer.

### IHC Staining

The primary antibodies anti-NCAPG (1:300, 24563-1-AP, Proteintech), anti-LEF1 (1:150, HA500273, HUABIO) and anti-SEMA7A (1:150, ER1905-75, HUABIO) were incubated at 4℃ for 16 h. The images were scanned with Panoramic Scanner (3DHISTECH Ltd, Budapest) and analyzed using CaseViewer 2.4 software (3DHISTECH Ltd. Budapest). Positive controls were established for sections, while samples incubated with primary antibodies were substituted with diluted universal antibody as negative controls. The IHC score was calculated as staining intensity × percentage of positive cells. The staining intensity was categorized as follows: 0 point for no yellow, 1 point for light yellow, 2 points for brown staining, and 3 points for dark brown particles. For positive cell percentage scoring: ˂25% positive cell received 0 point, 26%-50% positive cell received 1 point, 51%-75% positive cell received 2 points, and 76%-100% positive cell received 3 points. A high expression is defined a score >3 points, while a low expression is defined as a score ≤3 points.

### Cell culture and transfection

The human EC cell lines HEC-1-A, HEC-1-B and Ishikawa were purchased from Procell Life Science&Technology Co., Ltd, and were cultured following the provided instructions. Lentiviral particles for stable NCAPG knockdown and overexpression were purchased from Genechem (GeneChem Co., Ltd., Shanghai, China). The LEF1 shRNA, SEMA7A shRNA, and control shRNA were purchased from HonorGene Co., Ltd. All transfections were performed with Lipofectamine 2000 (Invitrogen), and stable cell lines were selected using puromycin (2.5 µg/µl) for 7 days.

### Quantitative real-time PCR (qRT-PCR)

Total RNA was extracted from cell lines with the TRIzol reagent (Takara, Shiga, Japan), and subsequently subjected to reverse transcription with the HiScript II Q RT SuperMix (Vazyme Biotech, China). qRT-PCR was performed using SYBR qPCR Master Mix (Vazyme Biotech, China) and Roche Light Cycler 96 (Roche Diagnostics, West Sussex, UK). Three biological replicates were utilized per intervention group, with beta-tubulin serving as a normalization reference. Relative mRNA levels were quantified using the comparative Ct (2-ΔΔCt) method. The sequences of qRT-PCR oligos used were as follows:

β-tubulin: 5'-GCTGCAAAACTTCTTCCCTCG-3' and 5'-TGTCCCTGTTCAAGGGAGTC-3'

NCAPG: 5'-TTCTGGCGCTTTCACGACT-3' and 5'-ACATGATAACACTGCCCGTCT-3'

LEF1: 5'-ACAAGGGACCCTCCTACTC-3' and 5'-ACCACGGGCACTTTATTT-3'

SEMA7A: 5'-GACCAGGGTGGGGAAAGT-3' and 5'-AGCCTGTTGAAGTTCTTGTTG-3'.

### Western blotting (WB) and Co-Immunoprecipitation (CoIP) assay

Total cell protein was extracted using cell protein extraction kit (xxx), and protein concentrations were determined using the BCA method (Pierce, Rockford, IL, USA). Equal protein quantities (30 µg per lane) were electrophoresed on the 10% SDS-PAGE gel and subsequently transferred to a polyvinylidene difluoride (PVDF) membrane. The membranes were then blocked with 5% bovine serum albumin (BSA) at room temperature for 2 h.

Incubation with the primary antibodies was performed overnight at 4 °C with anti-NCAPG antibody (1:2000, ab226805, Abcam), anti-LEF1 antibody (1:5000, 14927-1-AP, Proteintech), anti-SEMA7A antibody (1:2000, 18070-1-AP, Proteintech), anti-CDK6 antibody (1:5000, 14052-1-AP, Proteintech), anti-Cyclin E1 antibody (1:2000, 11554-1-AP, Proteintech), anti-Cyclin D1 antibody (1:5000, 26939-1-AP, Proteintech), anti-Cyclin D1 antibody (1:5000, 26939-1-AP, Proteintech), anti-Phospho-PI3 Kinase(1:1000, 4228T, Cell Signaling Technology), anti-PI3 Kinase (1:2000, 67071-1-Ig, Proteintech), anti-Phospho-AKT (1:2000, 4060T, Cell Signaling Technology), anti-AKT (1:2000, 10176-2-AP, Proteintech). The anti-β-tubulin antibody (1:5000, 10094-1-AP, Proteintech), anti-PCNA antibody (1:20000, 10205-2-AP, Proteintech) and anti-ATP1A1 antibody (1:5000, 14418-1-AP, Proteintech) were utilized as the internal control. Following washing, the membranes were incubated with appropriate HRP-secondary antibodies for 1 h at room temperature. Membranes were developed and detected by the enhanced chemiluminescence detection system (ChemiScope6100, China).

For IP assay, cells were washed twice with cold PBS and then incubated in lysis buffer (AWB0144, abiowell, China) for 30 minutes at 4 °C. The mixture was centrifuged at 12,000 rpm for 15 minutes. Subsequently, 200 µL of the protein sample was incubated with the appropriate primary antibody or normal rabbit IgG for 12 h at 4 °C with constant rotation. The sample was then mixed with 20 µL of Protein A/G agarose and centrifuged for 2 hours at 4 °C. The beads were washed three times with cell-lysis buffer, the captured immune complexes were analyzed by WB.

### Cell proliferation, migration and invasion assays

Following the manufacturer's instructions, the cell proliferation was measured using CCK8 (Abmole Bioscience, Shanghai, China). Cell migration ability was evaluated via the Transwell approach, and cell invasion was determined using a Matrigel-coated Transwell chamber.

### Assay for Transposase Accessible Chromatin with high-throughput sequencing (ATAC-seq) and RNA sequencing (RNA-seq)

The experimental procedure followed the protocol outlined in the ATAC-Seq Library Preparation Kit (https://www.apexbio.cn/atac-seq-library-preparation-kit.html). Quality control on the raw data was conducted using Trim Galore, and quality-filtered reads were subsequently mapped to the reference genome hg38. Genomic bedgraph visualization analysis was performed using IGV (Integrative Genomics Viewer), and motif enrichment analysis was performed using HOMER. For functional enrichment analysis, the clusterProfiler tool was employed to perform KEGG pathway annotation based on genes annotated by peaks in the differential analysis. RNA-seq analyses were carried out according to our previous study.

### Cell cycle analysis

Cells were rinsed with cold PBS and fixed with 75% ethanol for 24 h. They were then incubated with propidium iodide (PI) and RNase A for 30 minutes in the dark. CytoFLEX (BECKMAN COULTER, model A00-1-1102) flow cytometer was used to detect and analyze cell cycle distribution.

### Chromatin immunoprecipitation-qPCR Chromatin immunoprecipitation-qPCR (CHIP)

The JASPAR (http://jaspar.genereg.net/) bioinformatics tool predicts putative binding sites on promoter regions. The ChIP assay was performed using the Pierce agarose ChIP assay kit (ab500; Abcam) according to the manufacturer's protocols. The IgG antibody was used as a negative control. The ChIP signals were quantified via a quantitative PCR analysis on the Roche Light Cycler 96 (Roche Diagnostics, West Sussex, UK). The specific primer pairs for the SEMA7A promoter region are as follows: 5'-GGCTGGACTGCTCAGGTAA-3' and 5'-GAGGGAGGGTCAAAGCAG-3'.

### Luciferase assay

The pGL3-Basic Luciferase Reporter vector was purchased from HonorGene. The SEMA7 promoter was cloned into the pGL3-Basic Luciferase Reporter vector. Firefly and Renilla luciferase activities were measured using a Dual-Luciferase Reporter Assay System (Promega).

### Immunofluorescence (IF) staining

The cells were seeded onto 14 µm cell-climbing slices prior to reaching 70% confluence. They were then washed three times with PBS and fixed with 4% paraformaldehyde for 15 minutes, followed by permeabilization with 0.3% Triton at 37 °C for 30 minutes. Blocking was performed with 5% BSA in PBS for 1 h at 37 °C. Immunostaining was performed using the appropriate primary and secondary antibodies, and images were captured via a fluorescence microscope (BA210T, Motic).

### Xenografts in nude mice

The female athymic BALB/c nude mice (4-5 weeks of age) were obtained from Weitong Lihua Limited Company (Beijing, China) and housed under SPF conditions. HEC-1-A cells (2×10^6^ cells) were suspended in 100 µL PBS and then subcutaneously injected into the right armpit of nude mice. The width (W) and length (L) of the tumors were measured using a caliper every three days, and the volume (V) was calculated using the formula: V=0.5 × W2 × L. After 21 days, the mice were euthanized and the tumors were excised and weighted. All animal procedures were approved by the Ethics Committees of the Second Xiangya Hospital, with significant efforts made to minimize animal suffering.

### Statistical analysis

Chi-squared and Fisher's exact test were used to determine the association between IHC staining level and the clinicopathological characteristics of EC patients. A two-tailed Student's t-test calculated the statistical significance of differences between different groups. All statistical analyses were performed using Prism GraphPad version 9 (GraphPad Software, Inc., San Diego, CA, USA) and SPSS 26.0 software (IBM SPSS Statistics for Windows, Version 26.0). Statistical significance was set as P < 0.05.

## Results

### Integration analysis of ATAC-seq and RNA-seq

Our previous studies identified the biological functions of NCAPG in EC cells, demonstrating it promotes cell proliferation, migration, and invasion through the PI3K-AKT pathway. To uncover the intermediate mechanism by which NCAPG affects the PI3K-AKT pathway, we utilized stably transfected KD-NCAPG and KD-NC cells from the HEC-1-A cell line for ATAC-Seq, with two biological replicates for each sample set. Homer software was employed to predict motifs from all ATAC-Seq differential peak sequences (TSS±5KB). Concurrently, differentially down-regulated genes identified from previous RNA-Seq under the same conditions were used to obtain upstream transcription factors from the TRRUST database. These were then integrated with ATAC-Seq differential motif enrichment analysis to link chromatin accessibility changes with transcriptome differential expression profiles.

**Figure 1:**
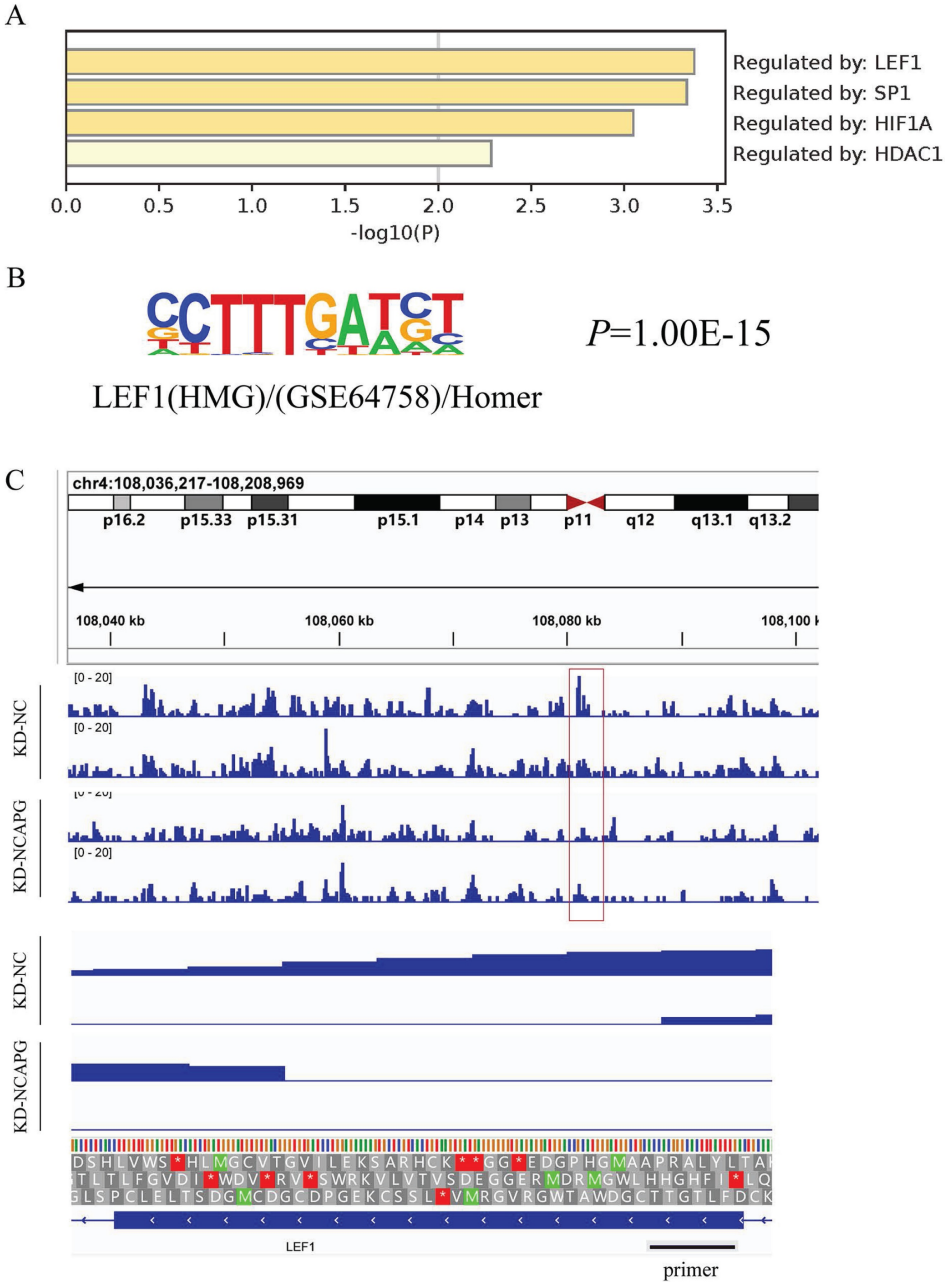
Effect of NCAPG on chromatin accessibility (A) Analysis of related transcription factors for downstream genes affected by NCAPG. (B) Motif annotation of ATAC-SEQ differential peaks between the NCAPG knockdown group and the control group revealed that the accessibility of LEF1 was significantly different. (C) Looking at the peak value of the chromatin site where LEF1 is located, it is found that after knocking down NCAPG, the amount of LEF1 that can bind is reduced.

Upstream transcription factors predicted by RNA-Seq results of down-regulated genes by knocking down NCAPG are shown in the Figure 1A, with LEF1 being the most effective regulatory factor. Motif annotation of differential peaks between ATAC-Seq groups revealed a P value for LEF1 of 1.00E-15, indicating altered chromatin accessibility between the NC group and the KD-NCAPG group (Fig. 1B). Further analysis showed that the peak value of LEF1 at the corresponding chromatin site after NCAPG knockdown, suggesting reduced LEF1 binding to chromatin (Fig. 1C). This indicates that knocking down NCAPG reduces the chromatin accessibility of LEF1, thereby diminishing the transcription of downstream genes regulated by LEF1.

Using the genes annotated by Peaks in the ATAC-Seq differential analysis, we performed KEGG functional enrichment analysis with clusterProfiler, where the PI3K/AKT pathway ranked first. This aligns with our previous study showing that the most relevant downstream pathway of NCAPG in EC cells is PI3K-AKT (Supplementary Fig. 1).

### NCAPG promotes EC cell proliferation, migration and invasion through LEF1

**Figure 2:**
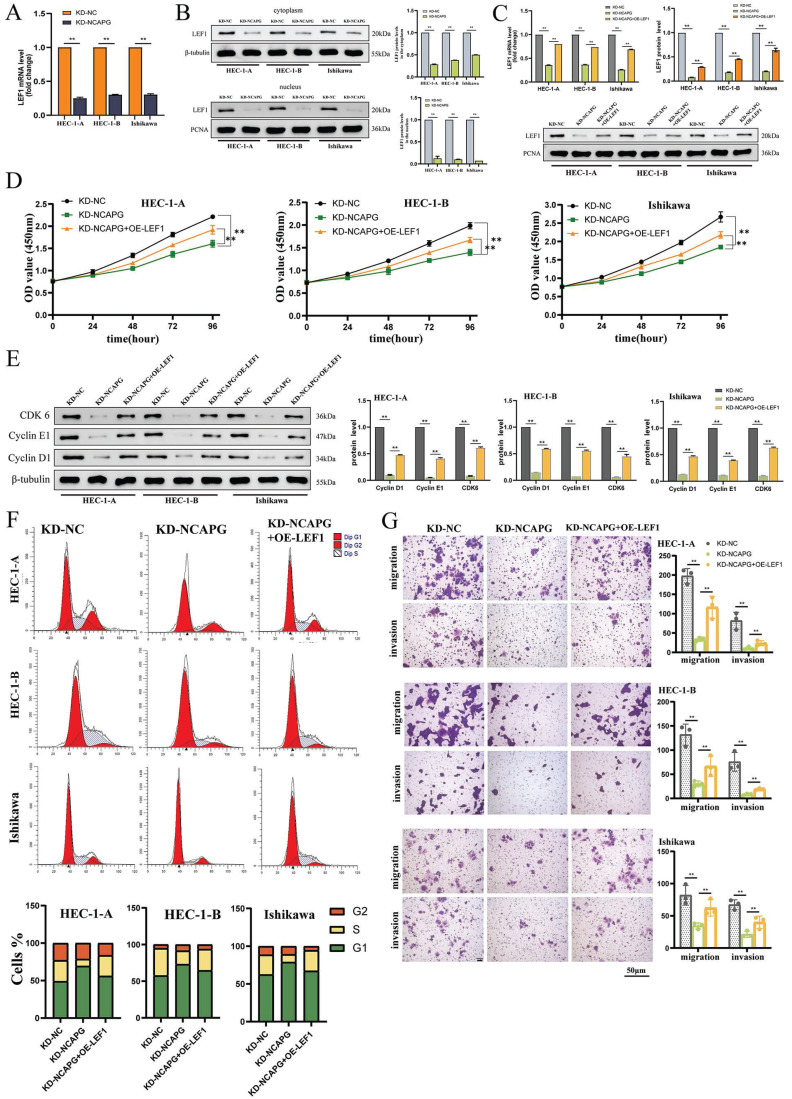
NCAPG regulates EEC cell proliferation, cell cycle, cell migration and invasion capabilities by affecting LEF1. (A) LEF1 mRNA expression is reduced after knocking down NCAPG. (B) After knocking down NCAPG, protein expression in both the nucleus and cytoplasm is reduced, and the reduction in the nucleus is more obvious. (C) Construction of a cell model overexpressing LEF1 after knocking down NCAPG. Overexpression of LEF1 in cell lines knocking down NCAPG can partially restore the inhibited proliferation (D), cell cycle G1-S transition (E, F), migration and invasion abilities (G).

The study examined the LEF1 mRNA levels in three NCAPG knockdown cell lines, analyzing nuclear and cytoplasmic proteins to understand the impact on LEF1 distribution within the cell. The findings, presented in Figure 2A and 2B, indicated a significant decrease in LEF1 mRNA expression and a notable reduction in LEF1 protein levels in both the cytoplasm and nucleus, with a more pronounced reduction in the nucleus. This suggests that decreasing NCAPG expression affects the nuclear entry of LEF1.

Next, LEF1 was overexpressed in NCAPG knockdown cell lines (Fig. 2C), and changes in cell proliferation, cell cycle, migration, and invasion functions were monitored. The results indicated that compared with the control group (KD-NC group), the NCAPG knockdown group (KD-NCAPG group) showed significant inhibition in cell proliferation, cell cycle G1-S transition, migration, and invasion abilities. However, overexpression of LEF1 in NCAPG-knockdown cell lines (KD-NCAPG+OE-LEF1 group) partially restored these initially inhibited functions, including proliferation, cell cycle G1-S transition, migration, and invasion (Fig. 2D-G). Thus, the findings suggest that NCAPG regulates EC cell proliferation, cell cycle, cell migration, and invasion capabilities by modulating LEF1.

### LEF1 binds to the promoter and activates SEMA7A

**Figure 3:**
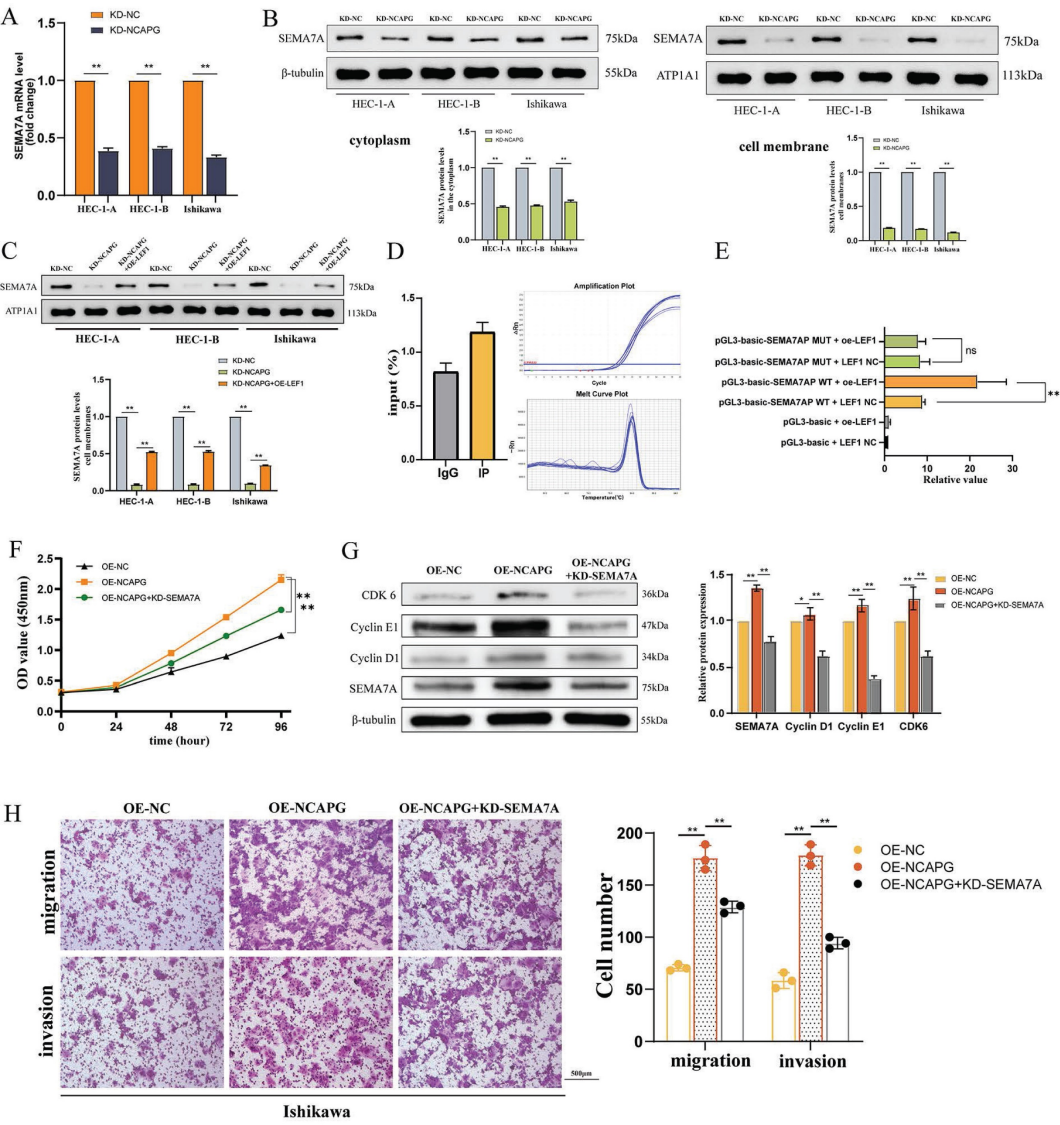
LEF1 transcriptionally regulates SEMA7A. (A) The mRNA expression of SEMA7A is reduced after knocking down NCAPG. (B) After knocking down NCAPG, the SEMA7A membrane protein decreases more obviously than the cytoplasmic protein. (C) Construction of a cell model overexpressing SEMA7A after knocking down NCAPG. (D) ChIP-qPCR indicates the binding of LEF1 and SEMA7A promoters. (E) After mutating the binding sites of LEF1 and SEMA7A, there is no change in the luciferase value of overexpressed LEF1. After knocking down SEMA7A, the accelerated cell proliferation (F), increased expression of cell cycle proteins in the G1-S phase (G), and enhanced migration and invasion abilities (H) caused by overexpression of NCAPG were reduced.

To identify downstream molecules regulated by NCAPG via the transcription factor LEF1, the study utilized the Metascape website for pathway enrichment analysis of down-regulated differential genes from RNA-Seq results (Fig. 3A). The analysis revealed enrichment in proliferation regulatory pathways. According to predictions from the JASPAR website, the promoters of LEF1 and SEMA7A possess binding sites (Supplementary Table 1).

Expression alterations of SEMA7A were assessed using qRT-PCR and WB across three cell lines following the knockidown of NCAPG, as depicted in the Figure 3B. After knocking down NCAPG, a significant reduction in SEMA7A mRNA and protein expression levels was observed, with a more pronounced decrease in protein levels localized to the membrane as compared to the cytoplasm. These findings confirmed that NCAPG positively regulate the expression of SEMA7A at both the mRNA and protein levels. Furthermore, the study employed an overexpression LEF1 plasmid to increase LEF1 expression in NCAPG knockdown cells and subsequently measured the resultant SEMA7A expression changes. The data revealed that t increased LEF1 expression partially restored the reduction in SEMA7A mRNA and protein expression induced by the knockdown of NCAPG, indicating that LEF1 can regulate SEMA7A expression (Fig. 3C).

We developed primers targeting the binding site of LEF1 and SEMA7A, and analyzed the binding of LEF1 to this site using ChIP-qPCR in the HEC-1-A cell line. The results showed that compared with the negative control IgG group, the IP group exhibited an average enrichment fold value of 1.45, implying that LEF1 protein binds to the predicted SEMA7A target region (Fig. 3D). Following this, the binding site of SEMA7A promoter associated with LEF1 was mutated, and vector plasmids for both wild-type and mutant SEMA7A were constructed. A dual-luciferase reporter assay was performed in the 293T cell line to identify the promoter activities of LEF1 and SEMA7A. The findings demonstrated that upon mutation of the SEMA7A promoter binding site, there was no significant difference in fluorescence values between the LEF1 overexpression group and the control group (Fig. 3E). These results indicate that LEF1 is instrumental in activating the promoter activity of SEMA7A.

Following the knockdown of SEMA7A in the Ishikawa cell line overexpressing NCAPG, the subsequent changes in proliferation, migration, and invasion ability of endometrioid endometrial cancer (EEC) cells were assessed. The results indicated that the enhanced cell proliferation, upregulated expression of cell cycle proteins in the G1-S phase, and the increased proliferation and invasion abilities driven by NCAPG overexpression were diminished upon SEMA7A knockdown. These results suggest that NCAPG exerts its influence on the biological behavior of EEC cells through downstream modulation of SEMA7A (Fig. 3F-H).

### SEMA7A binds to p85 protein and activates the PI3K-AKT pathway

**Figure 4:**
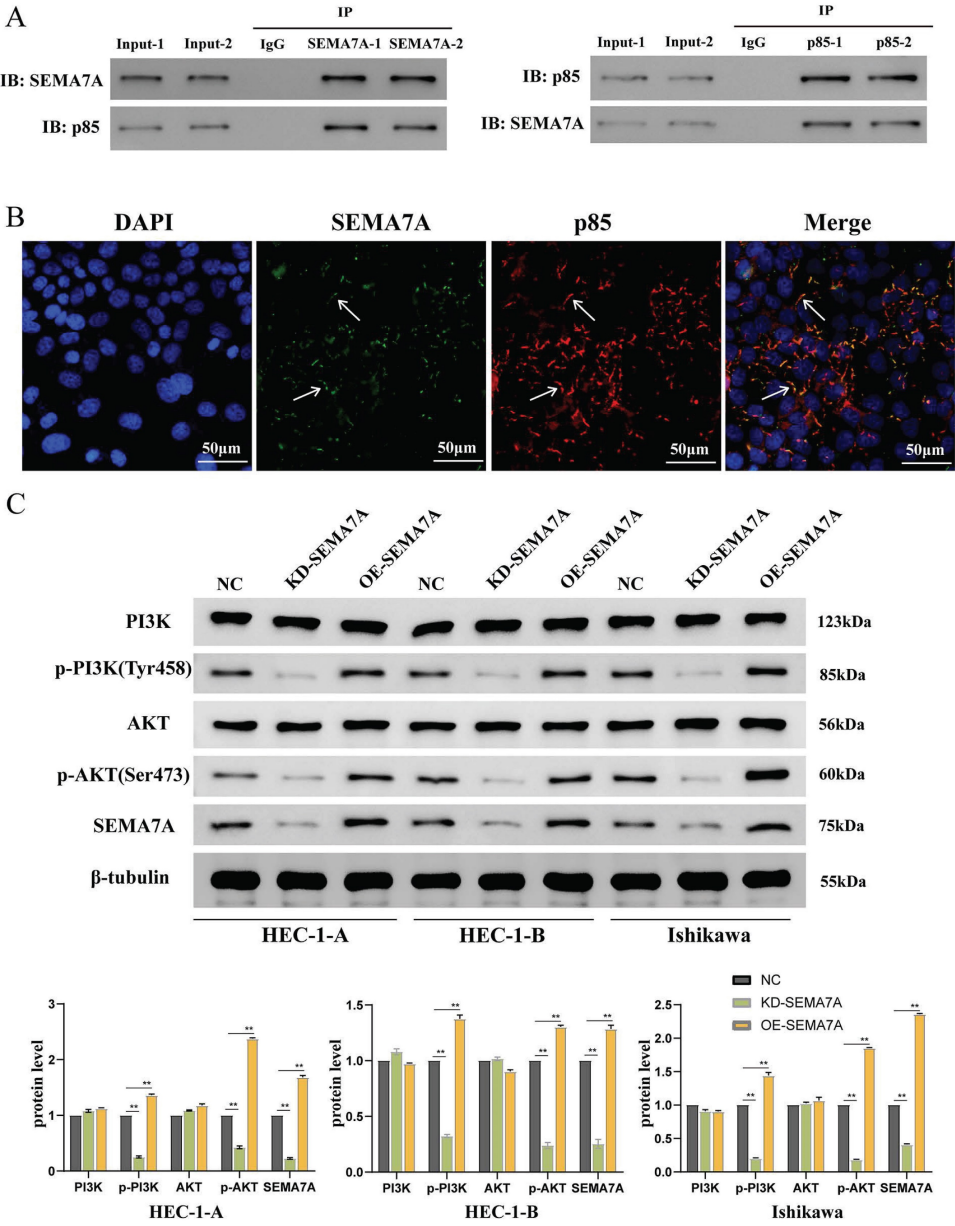
SEMA7A binds to the PI3K regulatory subunit p85 protein to activate the PI3K/AKT pathway. (A) CO-IP experimental results show that SEMA7A and p85 bind to each other. (B) Immunofluorescence co-localization experiment shows that SEMA7A and p85 are co-expressed on the cell membrane. (C) The expression of p-PI3K and p-AKT proteins is decreased after knocking down SEMA7A. On the contrary, the expression of p-PI3K and p-AKT proteins increased after overexpression of SEMA7A.

To identify potential intermediate molecules involved in SEMA7A-mediated regulation of the PI3K-AKT pathway, we investigated the transfection of a SEMA7A overexpression plasmid in the HEC-1-A cell line. The interaction between SEMA7A and p85 was verified using e Co-IP experiment conducted in duplicate. Our findings indicated that the SEMA7A antibody co-immunoprecipitated not only SEMA7A but also significantly enriched p85, whereas the p85 antibody co-immunoprecipitated both p85 and SEMA7A (Fig. 4A). Subcellular localization analysis via immunofluorescence further revealed that SEMA7A and p85 were co-localized on the cell membrane (Fig. 4B).

The changes in PI3K, AKT, and their phosphorylated forms p-PI3K and p-AKT were detected with either SEMA7A knocked down or overexpressed in cell lines. The results showed that knocking down SEMA7A led to decreased protein levels of p-PI3K and p-AKT, while the levels of PI3K and AKT remained relatively unchanged. Conversely, overexpression of SEMA7A resulted in increased expression of p-PI3K and p-AKT, without significantly affecting PI3K and AKT levels (Fig. 4C). These findings collectively suggest that SEMA7A activates the PI3K/AKT signaling pathway through interaction with the PI3K regulatory subunit p85.

### Effects of overexpression of LEF1 on transplanted tumors in nude mice

To examine the effect of LEF1 on cell proliferation *in vivo*, a nude mouse xenograft model was used. HEC-1-A cells from the KD-NC, KD-NCAPG, and KD-NCAPG+OE-LEF1 groups were subcutaneously injected into the flanks of nude mice. Mice were sacrificed after 21 days, and average tumor volume and body weight were measured.

**Figure 5:**
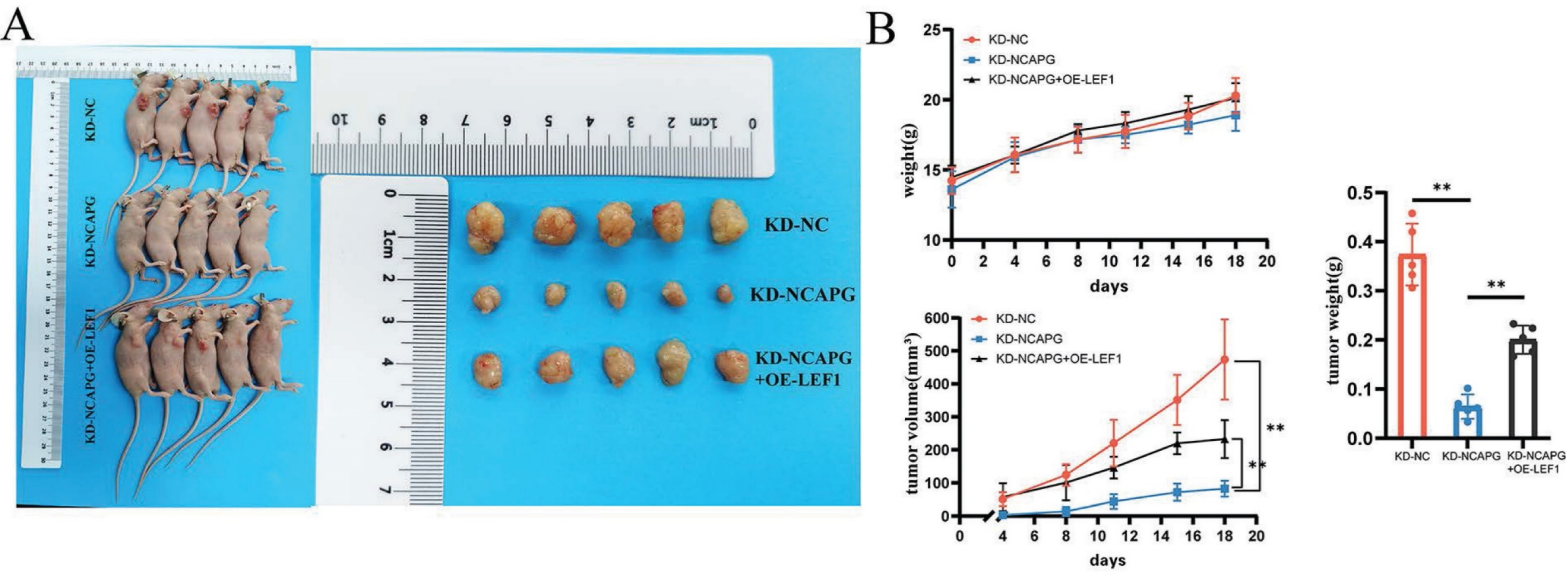
Effects of NCAPG, LEF1 and SEMA7A on transplanted tumors in nude mice. (A) Knockdown of NCAPG reduces the size of transplanted tumors in nude mice. Overexpression of LEF1 can partially restore the reduced size of transplanted tumors after knocking down NCAPG. (B) Comparison curves of nude mouse body weight changes, transplanted tumor volume measurements, and tumor *ex vivo* weight.

**Figure 6:**
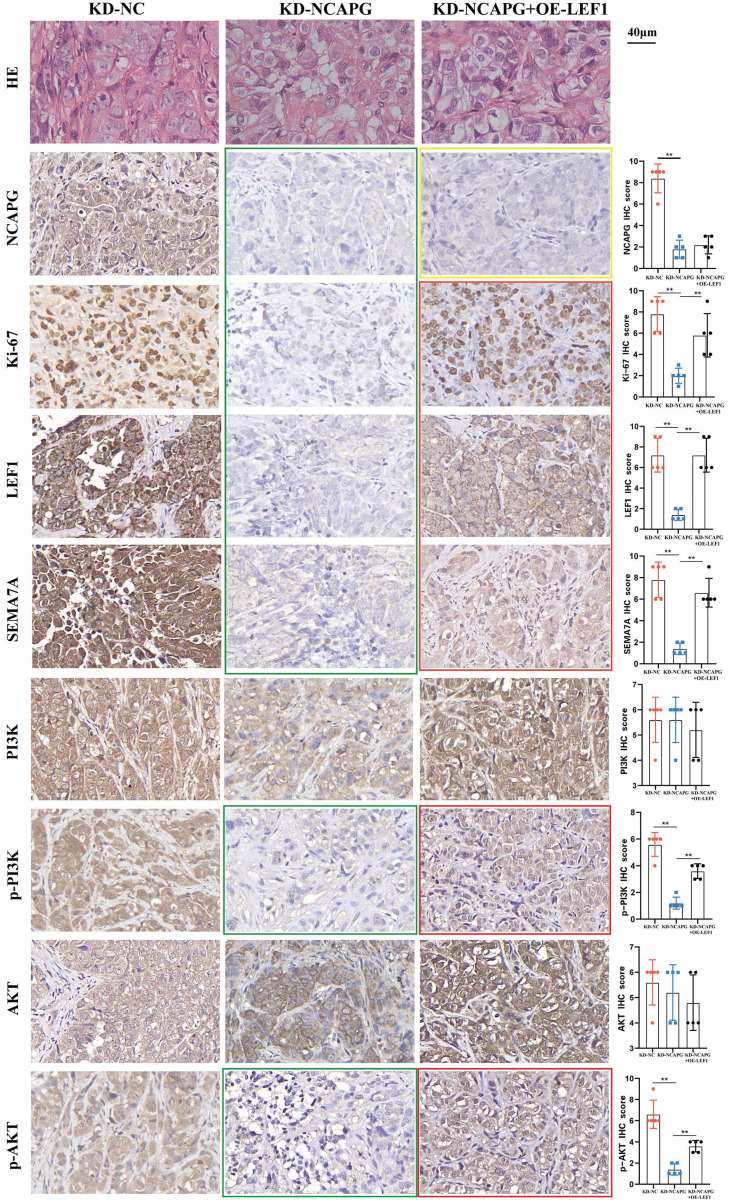
After knocking down NCAPG, the expression of NCAPG, Ki-67, LEF1, SEMA7A, p-PI3K and p-AKT decreased (green box). After overexpressing LEF1, the expression of NCAPG decreased compared with the KD-NC group, but remained unchanged compared with the KD-NCAPG group (yellow box). The expression of Ki-67, LEF1, SEMA7A, p-PI3K and p-AKT was partially restored compared with the KD-NCAPG group (red box).

The findings revealed that the tumor growth rate in the KD-NCAPG group was markedly reduced than that in the KD-NC group, while the tumor growth rate in the KD-NCAPG+OE-LEF1 group was partially restored compared to the KD-NCAPG group (Fig. 5A and B). IHC staining demonstrated that the expression levels of NCAPG, Ki-67, LEF1, and SEMA7A in the KD-NCAPG group were lower than those in the KD-NC group. Furthermore, within the KD-NCAPG+OE-LEF1 group, the expression levels of Ki-67, LEF1, and SEMA7A were lower than those observed in the KD-NC group but increased in comparison to the KD-NCAPG group. These data indicate that overexpression of LEF1 can restore the attenuated tumor growth after NCAPG knockdown *in vivo*. Additionally, levels of p-PI3K and p-AKT in the KD-NCAPG group were reduced relative to the KD-NC group, whereas their protein expressions were elevated in the KD-NCAPG+OE-LEF1 group when compared to the KD-NCAPG group (Fig. 6). It is suggested that the upregulation of SEMA7A, induced by increased LEF1 expression, can restore the down-regulation of the PI3K-AKT pathway following NCAPG knocking down.

### Expression correlation analysis of NCAPG, LEF1 and SEMA7A

We conducted IHC staining for LEF1 and SEMA7A on paraffin-embedded sections from 84 EEC patients. The analysis explored the correlation between LEF1 and SEMA7A expression and the clinicopathological characteristics of the patients. The results are detailed in Table 1 and Table 2.

Among the 84 EEC patients, there were 56 patients with high LEF1 expression, and 28 patients with low expression. Similarly, 56 patients exhibited high SEMA7A expression, with the remaining 28 showing low expression. Chi-square test analysis revealed that elevated expression of LEF1 was positively correlated with the patients' FIGO stage (P=0.048), pathological grade (P=0.024), myometrial invasion (P=0.000), and lymphatic vessel invasion (P=0.049). Additionally, high SEMA7A expression is positively correlated with patients' FIGO stage (P=0.002), pathological grade (P=0.024), myometrial invasion (P=0.000), and cervical stromal invasion (P=0.017).

The IHC protein expression results of NCAPG, LEF1, and SEMA7A were analyzed for consistency in the same batch of 84 patients, as shown in Table 3-5. Among them, 75 patients exhibited consistent expression levels of NCAPG and LEF1, with a McNemar test P-value of 1.000, indicating no difference in expression. The Kappa value for this consistency test was 0.757, indicating a high consistency between NCAPG and LEF1. Similarly, 69 patients displayed consistent expression levels of NCAPG and SEMA7A, with a McNemar test P-value of 1.000 and a Kappa value of 0.595, indicating no difference in expression and a high degree of consistency. Moreover, 78 patients had consistent expression levels of LEF1 and SEMA7A, with a McNemar test P-value of 1.000 and a Kappa value of 0.839, representing the highest degree of correlation. In summary, the IHC results from clinical patient samples demonstrate a positive correlation among the expressions of NCAPG, LEF1, and SEMA7A.

## Discussion

This study found NCAPG affects the chromatin accessibility of the transcription factor LEF1 via ATAC-Seq sequencing by regulating chromatin opening. LEF1 binds to the SEMA7A promoter in the cell proliferation-related pathways affected by NCAPG to promote its transcription. SEMA7A binds to the p85 protein, a regulatory subunit of PI3K, thus activating the PI3K/AKT pathway. Our results reveal that NCAPG exerts its oncogenic functions through the NCAPG/LEF1/SEMA7A/PI3K-AKT pathway in EC.

Previous studies on NCAPG in endometrial cancer were limited to bioinformatics analysis^15^, ^16^ or the confirmation of the effect of NCAPG on the *in vitro* growth of endometrial cancer cells by proliferation and invasion experiments^17^, ^18^. However, no studies have explored the mechanism by which NCAPG promotes cancer and whether it has the same effect *in vivo*. As a gene that promotes chromatin condensation and sister chromatid separation during the cell cycle, NCAPG may affect the genomic regions to which certain transcription factors bind through epigenetic modifications, thereby regulating downstream molecules and pathways.

In order to explore the specific downstream mechanism by which NCAPG promotes cancer, our study first employs ATAC-Seq to identify the open chromatin regions affected by NCAPG. ATAC-Seq uses the highly active Tn5 transposase to fragment chromatin and insert sequences containing PCR handles, which are used for amplification during subsequent library preparation to obtain accessible chromatin sites^19^. After processing and aligning ATAC-Seq fragments, Tn5 transposition enrichment of specific genomic regions is used to pinpoint accessible chromatin peaks in each sample, referred to as "ATAC-Seq peaks"^20^. ATAC-Seq peaks can be annotated with various transcription factor (TF) sequences, enabling comparison of chromatin accessibility signals across different sample types, and serve as the starting point for further downstream analyses^21^. Upon the ability of RNA-Seq to measure the gene expression accurately, the regulatory mechanism behind gene expression can be further elucidated through ATAC-Seq^19^. Thus, we conduct a differential analysis of the chromatin open state before and after knocking down NCAPG to identify regions with altered chromatin accessibility and TF binding. Meanwhile, the differential genes identified by RNA-Seq were analyzed to predict associated TFs, which were then intersected with TF sequences annotated by ATAC-Seq. This analysis revealed that LEF1 is the intermediate transcription factor regulated by NCAPG in downstream differential genes. In addition, KEGG pathway enrichment analysis of genes annotated by ATAC-Seq peaks after NCAPG knockdown identified the PI3K-AKT pathway as the most significantly altered. This further supports that NCAPG regulates the downstream PI3K-AKT pathway by altering chromatin accessibility.

Abnormal expression of LEF1 is related to tumorigenesis and proliferation, migration, and invasion of cancer cells^22^. In breast cancer, inhibition of LEF1 reduces phthalate-activated cell growth, invasion, and migration^23^. Increased expression of LEF1 in lung cancer cells enhances chemotactic invasion and cell growth^24^. In mouse models, LEF1 plays a role in endometrial development and gland bud formation, with protein expression returning to baseline when most glands are formed^25^. During proestrus in mice, elevated LEF1 levels promotes cell proliferation and gland formation. LEF1 is expressed in normal endometrium, its overexpression can be observed in the glandular component of human EC. In endometrial tumors with high LEF1 expression, the RNA expression of cyclin D1, a downstream target of LEF1, and the EC marker MMP7 increased 10-fold and 30-fold, respectively^25^. Our study found that knocking down NCAPG significantly reduced the mRNA and protein levels of LEF1, with a more pronounced decrease in the nucleus compared to the cytoplasm. This implies that NCAPG knockdown also hinders LEF1 nuclear entry. Additionally, overexpression of LEF1 partially restore the diminished cell proliferation, cell cycle progression, migration, and invasion abilities after NCAPG knockdown.

Further research is needed to identify downstream molecules regulated by LEF1 and participate in the PI3K/AKT pathway. Upon knocking down NCAPG, it was found to be enriched among the differential genes in the proliferation regulatory pathway. SEMA7A, which has a potential binding relationship with LEF1, showed decreased mRNA and protein levels after NCAPG knockdown. This reduction was more pronounced on the cell membrane than in the cytoplasm, suggesting that NCAPG predominantly affects the expression of SEMA7A on the cell membrane. SEMA7A belongs to the class VII signaling proteins of the semaphorin family^26^. It mainly drives tumor cell movement, adhesion, epithelial-mesenchymal transition (EMT), invasion, and metastasis. Additionally, SEMA7A promotes tumor-related angiogenesis, lymphangiogenesis, dendrite formation, and extracellular matrix remodeling, thereby altering the tumor microenvironment^27^-^29^.

The study constructed NCAPG knocked down as well as LEF1 overexpressed cell model, and discovered that LEF1 overexpression partially reverse the reduction in SEMA7A expression induced by NCAPG knockdown. ChIP-qPCR experiments confirmed that LEF1 binds to the promoter SEMA7A. Further, dual-luciferase experiments demonstrated that mutating the binding sites abolished LEF1's effect on SEMA7A expression, indicating that SEMA7A is a downstream target of LEF1. Knocking down SEMA7A partially reverse the enhanced cell proliferation, G1-S phase transition, and increased migration and invasion caused by NCAPG overexpression. Co-IP and IF co-localization experiments revealed that SEMA7A interacts with the PI3K regulatory subunit p85 on the cell membrane, and WB comfirmed that p-PI3K and p-AKT levels were positively correlated with SEMA7A.

In nude mouse transplanted tumors, overexpression of LEF1 reverses the tumor growth inhibition caused by NCAPG knockdown. IHC staining showed that after knocking down NCAPG and overexpressing LEF1, the expression of the downstream proteins SEMA7A, p-PI3K, and p-AKT was partially restored. Examining the expression levels of LEF1 and SEMA7A in the EEC patients and the clinical pathological correlation among 84 patients, the high expression ratio of LEF1 and SEMA7A was 66.7%, close to 67.9% of NCAPG. High LEF1 and SEMA7A expression positively correlates with the patients' FIGO stage, pathological grade, and myometrial invasion. Correlation test showed a strong positive correlation between NCAPG with LEF1 and SEMA7A.

**Figure 7:**
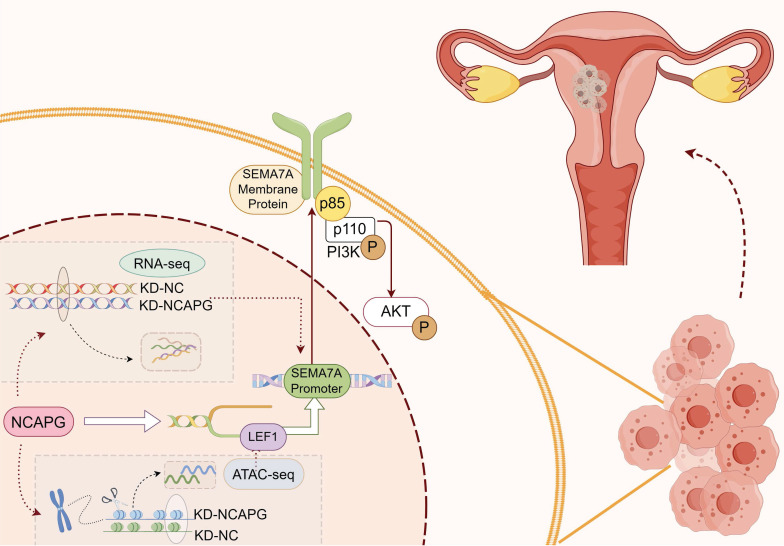
Diagram of the biological role of NCAPG in endometrioid cancer. NCAPG first regulates the binding of the transcription factor LEF1 to chromatin, and LEF1 promotes its transcription by interacting with the SEMA7A promoter. The generated membrane protein SEMA7A binds to the p85 protein, a regulatory subunit of PI3K, activating the PI3K/AKT pathway and promoting the proliferation, migration and invasion of endometrioid cancer cells. By Figdraw.

In summary, this study confirms that NCAPG promotes SEMA7A transcription in EEC cells by regulating the accessibility of the transcription factor LEF1. SEMA7A binds to the PI3K regulatory subunit p85, thereby activating the PI3K-AKT pathway, which in turn promotes cell proliferation, migration, and invasion (Fig. 7).

## Conclusion

In summary, this study confirms that NCAPG promotes SEMA7A transcription in endometrioid cancer cells by regulating the accessibility of the transcription factor LEF1. SEMA7A binds to the PI3K regulatory subunit p85 to activate the PI3K/AKT pathway, promoting proliferation, migration, and invasion function (Fig. 7 and Supplementary Table 2).

## Supplementary Material

Supplementary figure and table.

## Figures and Tables

**Table 1:** Correlation between LEF1 expression and clinicopathological parameters in 84 patients with endometrial cancer.

Characteristics		Expression of LEF1		
	Total	High (%) (n=56)	Low (%) (n=28)	P value
Menopausal status				
Postmenopausal	56	41	15	0.072
Premenopausal	28	15	13	
FIGO Stage				
I	58	34	24	0.048*
II	14	11	3	
III	12	11	1	
Grade				
G1+G2	66	40	26	0.024*
G3	18	16	2	
Myometrial invasion				
<1/2	59	32	27	0.000*
≥1/2	25	24	1	
Cervical stromal invasion				
Absent	65	41	24	0.197
Present	19	15	4	
Lymph node metastasis				
Absent	74	47	27	0.154
Present	10	9	1	
Lymphovascular invasion				
Absent	68	42	26	0.049*
Present	16	14	2	
Mismatch repair status				
proficient	57	37	20	0.254
deficient	9	8	1	
No data	18			

*Statistically significant. FIGO: Federation of International of Gynecologists and Obstetricians.

**Table 2:** Correlation between SEMA7A expression and clinicopathological parameters in 84 patients with endometrial cancer.

Characteristics		Expression of SEMA7A		
	Total	High (%) (n=56)	Low (%) (n=28)	P value
Menopausal status				
Postmenopausal	56	42	14	0.022*
Premenopausal	28	14	14	
FIGO Stage				
I	58	32	26	0.002*
II	14	13	1	
III	12	11	1	
Grade				
G1+G2	66	40	26	0.024*
G3	18	16	2	
Myometrial invasion				
<1/2	59	32	27	0.000*
≥1/2	25	24	1	
Cervical stromal invasion				
Absent	65	39	26	0.017*
Present	19	17	2	
Lymph node metastasis				
Absent	74	47	27	0.090
Present	10	9	1	
Lymphovascular invasion				
Absent	68	43	25	0.169
Present	16	13	3	
Mismatch repair status				
proficient	57	40	17	1
deficient	9	6	3	
No data	18			

*Statistically significant. FIGO: Federation of International of Gynecologists and Obstetricians.

**Table 3:** Concordance analysis between NCAPG and LEF1 expression.

	Expression of NCAPG				
Expression of LEF1	High	Low	Total	McNemar	Kappa
High	52	4	56	P=1.000	0.757
Low	5	23	28		
Total	57	27	84		

**Table 4:** Concordance analysis between NCAPG and SEMA7A expression.

	Expression of NCAPG				
Expression of SEMA7A	High	Low	Total	McNemar	Kappa
High	49	7	56	P=1.000	0.595
Low	8	20	28		
Total	57	27	84		

**Table 5:** Concordance analysis between LEF1 and SEMA7A expression.

	Expression of LEF1				
Expression of SEMA7A	High	Low	Total	McNemar	Kappa
High	53	3	56	P=1.000	0.839
Low	3	25	28		
Total	56	28	84		
