# Supplementary material for: NCAPG promotes the malignant progression of endometrioid cancer through LEF1/SEMA7A/PI3K-AKT
Source: J Cancer. 2025 Jan 1;16(2):445–59. doi: 10.7150/jca.100951 (PMC11685698; doi:10.7150/jca.100951)
Supplement: Supplementary file 1 — Supplementary figure and table. [file jcav16p0445s1.pdf]

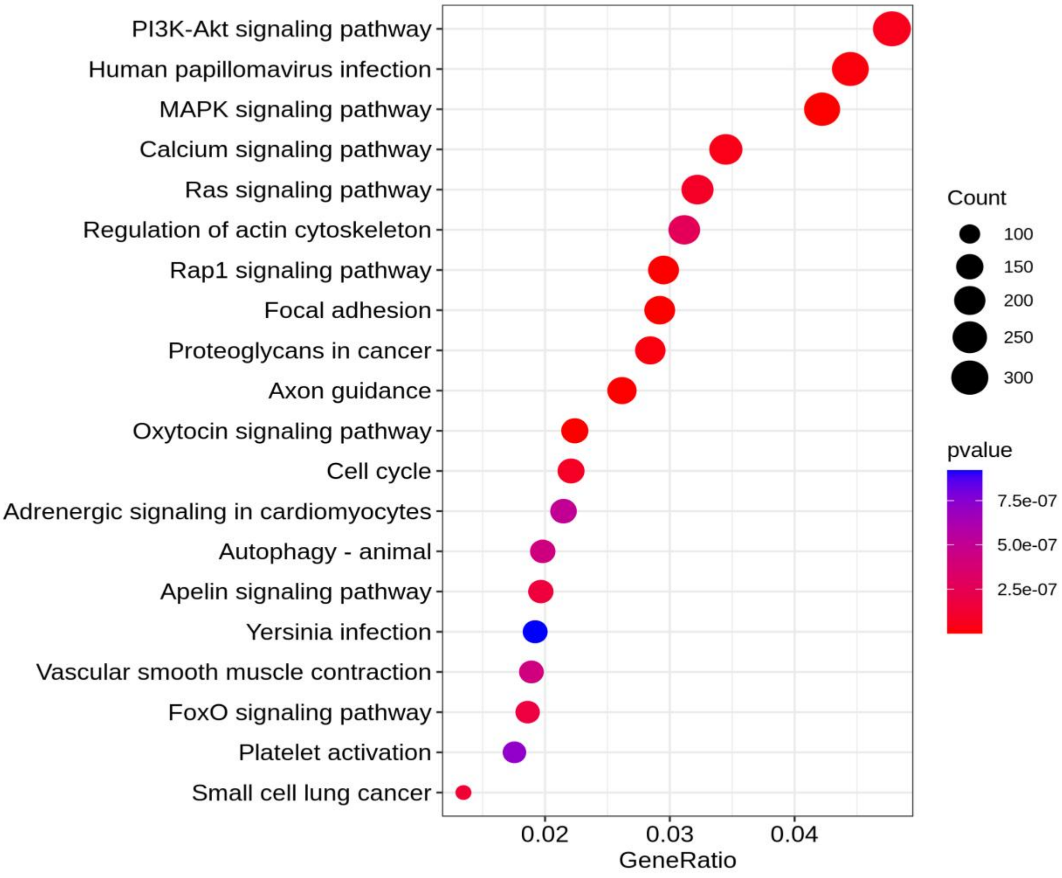

Supplementary Fig.1 KEEG enrichment results of differential genes between ATAC-Seq groups after knocking down NCAPG. The PI3K/AKT pathway ranks first.

Supplementary Table 1 The binding site of LEF1 and SEMA7A promoter was predicted from JASPAR.

Matrix ID	Name	Score	Relative score	Start	End	Strand	Predicted sequence
MA0768.1	LEF1	8.229587	07981	1482	1496	+	CAAGATGAAAAATTT

Supplementary Table 2 NCAPG biochemical and molecular characterization

Regulate	Biological effects
LEF1	Affects its binding to chromosomes
SEMA7A	Affects its conversion through LEF1
PI3K-AKT	Affects activation through SEMA7A
proliferation*	Promote
Invasion*	Promote
metastasis*	Promote

*Works both in vivo and in vitro.
